# Brain natriuretic peptide and all-cause mortality in patients treated with haemodialysis

**DOI:** 10.1186/s12882-025-04251-8

**Published:** 2025-06-23

**Authors:** Maria K Svensson, Rita Nassar, Jan Melin, Magnus Lindberg, Hans Furuland, Jenny Stenberg

**Affiliations:** 1 Department of Medical Sciences, Renal Medicine, Uppsala University Hospital, Akademiska sjukhuset, Entrance 40, floor 5, Uppsala, SE 751 85 Sweden; 2 Uppsala Clinical Research Centre, Uppsala, Sweden; 3 Faculty of Health and Occupational Studies Department of Caring Sciences, University of Gävle, Gävle, Sweden

**Keywords:** Brain natriuretic peptide, Fluid overload, Haemodialysis, Survival analysis

## Abstract

**Background:**

Brain natriuretic peptide (BNP) is a hormone secreted from the heart in response to fluid overload. In patients with chronic kidney disease (CKD), inadequate fluid management during haemodialysis may cause fluid overload and overhydration (OH), risk factors for mortality. The aim of this exploratory pilot study was to analyse the relationships between BNP, OH and all-cause mortality in patients with CKD and haemodialysis.

**Methods:**

In this prospective observational study, five-year survival was analysed in 64 patients with CKD and haemodialysis. Bivariate correlations were performed to analyse the relationships between BNP, OH, and all-cause mortality. Cox regression analysis was performed to adjust the relationship between BNP and all-cause mortality for selected clinical and biochemical characteristics, collected at baseline.

**Results:**

By the end of the study, 33 patients (52%) had died. In bivariate correlation analysis age (*r* = 0.38), BNP (*r* = 0.48), handgrip strength (*r*=-0.34), lean tissue index (*r*=-0.41) and CRP level (*r*=-0.34, *p* = 0.007) were significantly associated with all-cause mortality. In a linear regression model, BNP was found to be a significant predictor of all-cause mortality (HR 2.61). However, after adjusting for age, handgrip strength, and CRP, BNP was no longer a statistically significant predictor of all-cause mortality. Instead, age, handgrip strength and CRP were significant predictors of all-cause mortality (HR 1.04; HR 0.95 and HR 2.61, respectively).

**Conclusions:**

In this study, BNP was correlated with all-cause mortality in patients with CKD and haemodialysis, but OH was not. When adjusting for other clinical and biochemical factors, age, inflammation, and handgrip strength were found to be independent and more important predictors of all-cause mortality than BNP.

**Supplementary Information:**

The online version contains supplementary material available at 10.1186/s12882-025-04251-8.

## Introduction

Chronic kidney disease (CKD) is associated with a significantly increased risk of cardiovascular (CV) mortality, 10 to 20 times greater risk than the general population [1, 2]. One key contributor to this elevated risk is overhydration (OH), a common condition in patients with kidney failure treated with haemodialysis. Approximately 25% of these patients present with OH of 2.5 L or more before dialysis [3, 4, 5], and OH has been linked to reduced survival [5, 6, 7, 8, 9, 10, 11, 12]. Effective volume management therefore is a critical aspect of care for patients with kidney failure and haemodialysis.

Traditionally, fluid status is assessed clinically [13], but in the last decade the use of bioimpedance techniques for assessment of fluid status has increased [14]. However, given the high prevalence of OH and its significant impact on patient outcomes, there is a need for more accurate and reliable methods identifying and managing OH. Brain natriuretic peptide (BNP) is a hormone produced by the cardiomyocytes in heart in response to fluid overload, and elevated levels of BNP and NT-proBNP are associated with increased morbidity and mortality [15, 16]. Previous studies have demonstrated an intraindividual correlation between BNP levels and OH in patients with kidney failure and haemodialysis treatment [17, 18].

Considering the critical importance of volume management in patients with kidney failure and haemodialysis, understanding the role of biomarkers such as BNP in predicting fluid status and its relation to patient outcomes is essential. The aim of this exploratory pilot study was therefore to prospectively assess the relationships among BNP, OH, and all-cause mortality in patients with kidney failure undergoing haemodialysis treatment.

## Materials and methods

### Study population

**Fig. 1:**
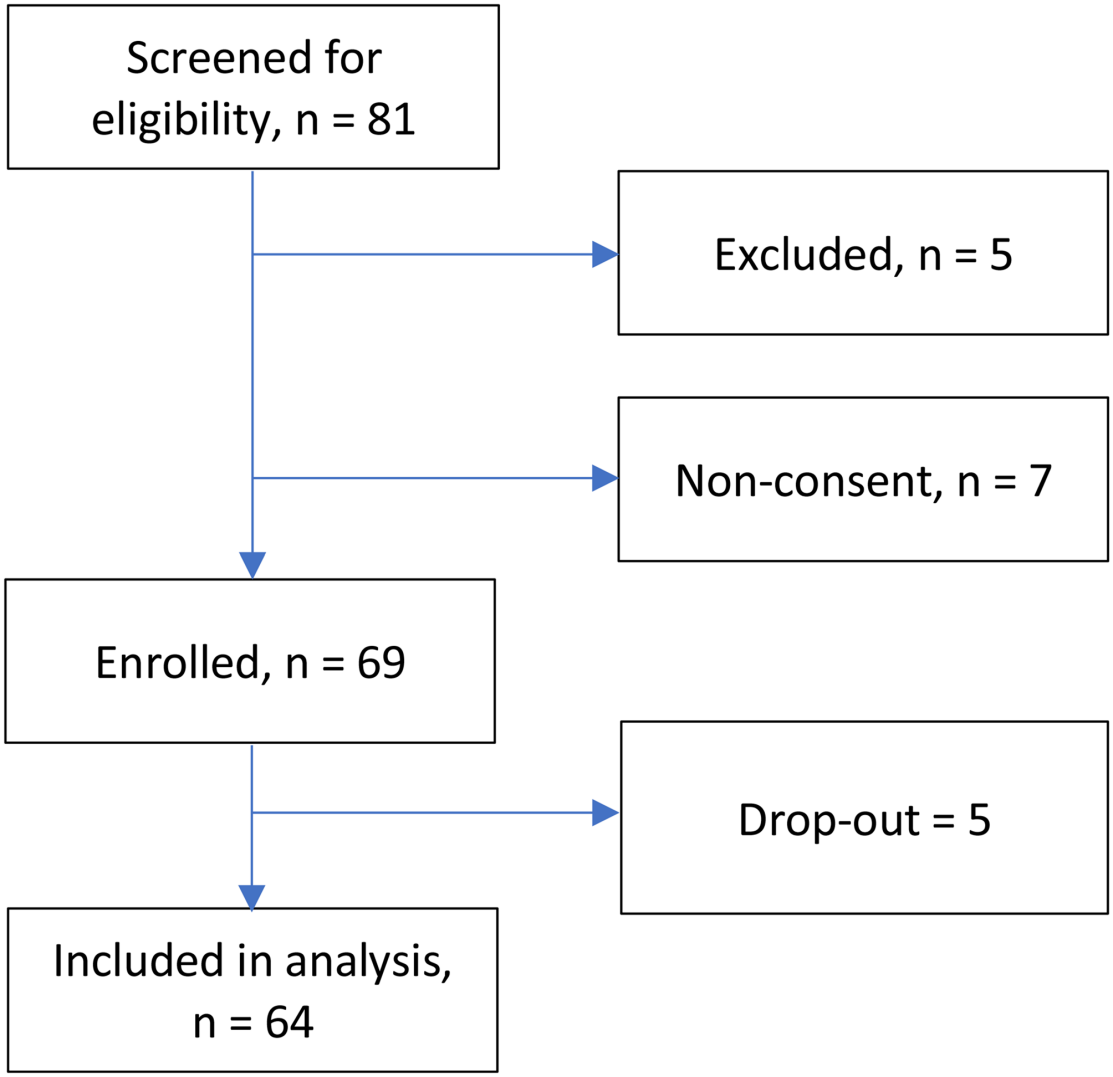
Study participants inclusion procedure. Drop-outs were due to renal transplantation (*n* = 1), recovered renal function (*n* = 1), conversion to peritoneal dialysis (*n* = 1), or death prior to data collection (*n* = 2)

Study inclusion criteria were treatment with intermittent haemodialysis for ≥ 3 months, age ≥ 18 years and ability to provide informed consent. The exclusion criterion was having a unipolar pacemaker, as this was considered incompatible with bioimpedance measurements at the time. Analyses are based on data from 64 subjects (Fig. 1). All study procedures were performed in accordance with the principles of the Declaration of Helsinki, and all study participants had provided written informed consent prior to data collection. The study protocol was approved by the Regional Ethical Review Board in Uppsala, Sweden (dnr 2017/006).

### Data collection

At baseline, between March and June 2017, collection of blood samples, blood pressure measurements and bioimpedance measurements were performed prior to a regular planned midweek dialysis session. BNP assays were performed using an Alere Triage Meter-Pro (Alere Inc., DE, USA), which enables bedside assessment. BNP, rather than NT-proBNP was chosen for analysis, since BNP levels in dialysis patients differ less from non-dialysis subjects and are less affected by dialysis treatment [19, 20, 21, 22]. To assess hydration status, the body composition monitor (BCM; Fresenius Medical Care, Bad Homburg, Germany) was used. Blood pressure and heart rate were measured with the blood pressure monitor integrated in a Fresenius 5008 haemodialysis machine (Fresenius Medical Care, Bad Homburg, Germany). Information about medical history, treatment modalities, additional biomarkers, and nutritional status including handgrip strength, was collected from medical records. At the clinic where the study was performed a hand dynamometer is used to evaluate the patients isometric grip force (handgrip strength) on a regular basis. Patients perform the test prior to a dialysis treatment, twice or three times annually, in a standing or sitting position and the best value out of three attempts is registered in the patient’s medical record.

Five years after baseline, in March 2022, information on changes in treatment modalities and mortality – date and cause of death, was extracted from the death certificate in the electronic healthcare records. During the follow up period no additional data were collected. However, at end of study, comorbidities were assessed retrospectively, using a modified version of Charlson comorbidity index (CCI), in which each comorbidity, but not age, is weighted and the total CCI is scored from 0 to 29, a higher score indicating a higher comorbidity burden [23].

### Data analysis

For analysis, to further explore the effects of BNP and OH, the study participants were divided into four groups according to their BNP levels and fluid status (OH) at baseline. Based on the literature, high BNP was defined as BNP ≥ 500 pg/ml [18], and fluid overload was defined as bioimpedance-measured OH ≥ 2.5 L [7, 24]:

•Group A (low BNP, low OH).
•Group B (low BNP, high OH).
•Group C (high BNP, low OH).
•Group D (high BNP, high OH).

The baseline characteristics of the participants were summarized using descriptive statistics. Normally distributed variables are presented as the means with standard deviations (SD), and non-normally distributed variables are presented as medians and interquartile ranges (IQR). Categorical variables are expressed as frequencies (n) and percentages (%). Given the positively skewed distributions of the BNP and CRP values, log-transformation (Log^10^) of these variables were performed. Comparisons between the four groups described above, were made using Kruskal-Wallis ANOVA or Chi2, and the Mann-Whitney U test was used for post hoc analysis investigating pairwise differences between the groups. To analyse linear relationships, the Spearman correlation coefficient was used.

Time-to-event (survival) was calculated in months from the start to the end of the study for all participants. However, for patients who underwent kidney transplantation, their data were censored at the date of transplantation. To compare survival between groups, the Kaplan‒Meier log-rank test was used. Additionally, a Cox regression analysis was performed to adjust the relationship between BNP and all-cause mortality for selected baseline clinical and biochemical characteristics, that in addition to BNP were found to correlate to all-cause mortality in the bivariate analysis. The number of variables analysed in the regression model was adjusted to the number of deaths, with an event-to-variable ratio of 10:1. Multicollinearity among the covariates was assessed using variance inflation factors (VIFs). A VIF > 5 was considered indicative of significant multicollinearity. The level of significance was set to *p* < 0.05, and the statistical analyses were performed via IBM SPSS Statistics version 28.0.

## Results

### Clinical and biochemical characteristics of the study participants

The study participants (*n* = 64) were 70 ± 13 years old and had been treated with haemodialysis for 37 (16–75) months on average. 77% were men, and most patients (72%) were treated with online haemodiafiltration (HDF). At baseline 47% had diabetes, type 1 or 2. The median BNP was 365 (178–833) pg/ml, and the mean OH was 2.2 ± 1.4 (1.2–3.2) L. The median overall CCI (retrospectively assessed at follow-up) was 7 (5.25-8), and during the five-year follow-up nine patients (14%) underwent kidney transplantation. The clinical and biochemical characteristics of all the study participants are presented in Table 1. Differences between deceased and alive study participants are shown in Table 2 and the underlying kidney diseases in Supplementary Fig. 1.

**Table 1:** Clinical and biochemical characteristics of the study participants overall and in the four subgroups based on the BNP level and hydration status (OH) at baseline (*n* = 64)

	All Participants (*n* = 64)	Group A Low BNP Low OH (*n* = 29)	Group B Low BNP High OH (*n* = 11)	Group C High BNP Low OH (*n* = 12)	Group D High BNP High OH (*n* = 12)	*P*-value
Men/Women (n, %)	49 (77)/15(23)	20 (69)/9 (31)	11 (100)/0 (0)	8 (67)/4 (33)	10 (83)/2 (17)	0.15
Age (years)	70 ± 13	**66 ± 12** ^**C**^	**62 ± 18** ^**C**^	**80 ± 7** ^**A, B**^	73 ± 11	**0.003**
Body weight pre-HD (kg)	82.4 ± 18	84 ± 22	82 ± 20	76 ± 18	76 ± 16	0.20
Time on dialysis (months)	37 (16–75)	33 (18–67)	44 (20–92)	55 (23–82)	28 (9–74)	0.59
Hours/treatment	4.5 (4–4.5)	4.5 (4–4.5)	4.8 (4–5)	4.2 (4–4.5)	4.2 (4–4.5)	0.11
Treatments/week	3 (3–3)	3 (3–3)	3 (3–3)	3 (2–3)	3 (3–3)	0.16
Dialysis modality, HDF (n, %)	46 (72)	22 (76)	10 (91)	7 (58)	7 (58)	0.22
SBP (mmHg)	144 ± 26	145 ± 28	141 ± 29	139 ± 28	149 ± 20	0.70
DBP (mmHg)	67 ± 16	69 ± 15	65 ± 20	59 ± 17	70 ± 13	0.33
**Comorbidities**						
Diabetes type 1/2 (n, %)	5 (8)/25 (39)	2 (7)/8 (28)	2 (18)/4 (36)	1 (8)/6 (50)	0 (0)/7 (58)	0.35
IHD (n, %)	19 (30)	7 (24)	4 (36)	5 (42)	3 (25)	0.66
Other heart disease (n, %)	21 (33)	11 (38)	1 (9)	3 (25)	6 (50)	0.17
CCI	7 (5.25-8.0)	7 (5.0-8.5)	8 (4.0–10.0)	7 (6.25–7.75)	7 (6.0-8.75)	0.86
**Laboratory test results**						
Haemoglobin (g/L)	109 ± 13.4	**114 ± 11.8** ^**D**^	**112 ± 14.0** ^**D**^	106 ± 11.7	**97.5 ± 11.0** ^**A, B**^	**0.003**
Albumin (g/L)	30.3 ± 4.3	**31.7 ± 3.4** ^**C, D**^	**32 ± 4.0** ^**D**^	**29 ± 3.4** ^**A**^	**26 ± 4.5** ^**A, B**^	**0.001**
Phosphate (mmol/L)	1.5 ± 0.5	1.5 ± 0.6	1.3 ± 0.4	1.6 ± 0.6	1.5 ± 0.4	0.56
BNP (pg/ml)	365 (178–833)	204 (118–275)	268 (73–406)	1035 (824–1255)	1440 (652–3877)	NA
CRP (mg/L)	7.0 (2.7–18)	4.7 (2–17)	4.5 (1.4–18)	11 (3–20)	15.5 (3.8–28)	0.24
**Volume status**						
OH (L)	2 (1.2–3.2)	1.5 (0.9–2.0)	3.4 (3-0-3.7)	1.9 (1.0-2.1)	4.5 (2.7–4.7)	NA
NH weight (kg)	80.1 ± 18.4	86 ± 17.9	78.9 ± 19.9	74.6 ± 18.0	72.6 ± 15.5	0.10
Target weight (kg)	80.1 ± 18.6	85 ± 17.7	80 ± 22.0	75 ± 20.0	74 ± 15.7	0.26
UFV (L)	2.1 (0.9–2.7)	1.4 (0.7–2.8)	2.6 (0.8–2.9)	2.1 (0.9–2.4)	2.5 (0.8–2.7)	0.26
**Nutritional status**						
Handgrip (kg)	24 (20–36)	24 (20–36)	**33 (24–41)** ^**C**^	**19 (16–26)** ^**B**^	22.5 (21–33)	**0.04**
BMI (kg/m^2^)	27.3 ± 5.4	28.7 ± 5.2	25.5 ± 6.0	25.7 ± 5.6	25 ± 3.7	0.13
LTI (kg/m^2^)	11.6 ± 2.5	**12.3 ± 2.6** ^**C**^	**12.7 ± 1.6** ^**C**^	**9.5 ± 1.5** ^**A, B**^	11 ± 2.5	**0.002**
**Mortality**						
All-cause mortality (n, %)	33 (52)	12 (41)	4 (36)	10 (83)	7 (58)	0.063
CV mortality (n, %)	15 (46)	9 (75)	1 (25)	3 (30)	2 (29)	0.47

The data are expressed as the means ± SDs, medians (IQRs) or frequencies (percentages), as appropriate

BMI: body mass index; BNP: brain natriuretic peptide; CCI: Charlson comorbidity index; CV: cardiovascular; CRP: C-reactive protein; DBP: diastolic blood pressure; IHD: ischaemic heart disease; LTI: lean tissue index; NH: normal hydration; OH: overhydration; SBP: systolic blood pressure; UFV: ultrafiltration volume; UFR: ultrafiltration rate. Comparisons between all four groups were made with Kruskal-Wallis H or Chi^2^ as relevant. Letter in superscript indicates pairwise significant difference to the group represented by the letter, as analysed with Mann-Whitney U test. *P* < 0.05 was considered significant and in bold

**Table 2:** Clinical and biochemical characteristics of the study participants deceased or alive at end of study (*n* = 64)

	Deceased, *n* = 33	Alive, *n* = 31	*p*-values
Men/Women (n, %)	25 (77)/8 (23)	24 (76)/7 (24)	0.88
Age (years)	75 ± 9	63 ± 14	**0.003**
Time on dialysis (months)	48 (23–86)	26 (15–56)	**0.04**
CCI	7 (7–9)	7 (5–8)	**0.02**
CRP (mg/L)	11 (3.8–27)	4 (1.8–15)	**0.008**
Haemoglobin (g/L)	109 ± 13	110 ± 14	0.49
Albumin (g/L)	29 ± 4	31 ± 4	**0.06**
BNP (pg/ml)	581 (305–1230)	191 (95–471)	**< 0.001**
SBP (mmHg)	135± 29	153 ± 20	**0.006**
DBP (mmHg)	61± 15	73 ± 15	**0.006**
OH (L)	2 (1.3–2.8)	2.1 (1.2–3.6)	0.73
HGS (kg)	22 (18–30)	30 (21–41)	**0.009**
BMI (kg/m^2^)	27 ± 5	27 ± 6	0.98
LTI (kg/m^2^)	11 ± 2	13 ± 3	**0.001**

The data are expressed as the means ± SDs, medians (IQRs) or frequencies (percentages), as appropriate

BMI: body mass index; BNP: brain natriuretic peptide; CCI: Charlson comorbidity index; CRP: C-reactive protein; DBP: diastolic blood pressure; LTI: lean tissue index; OH: overhydration; SBP: systolic blood pressure. Differences between deceased and alive study participants analysed using Mann-Whitney U-test. *P* < 0.05 was considered significant and in bold

When the study participants were divided into four groups based on their BNP level (≥ 500 or < 500 pg/ml) and hydration status measured by bioimpedance (OH ≥ 2.5 L or < 2.5 L), the largest group of patients (45%) was found to have low BNP and low OH (group A). In this group, the median BNP value was 204 pg/ml; in group B (low BNP, high OH), the median BNP was 268 pg/ml; in group C (high BNP, low OH), the median BNP value was 1035 pg/ml; and in group D (high BNP, high OH), the median BNP value was 1440 pg/ml. OH was highest in group D (4.5 L). Patients in groups B and D, with OH values above the cut-off of 2.5 L, also reported more symptoms related to fluid overload (FO) (data not shown). Patients in groups C, with BNP values above the cut-off, 500 pg/ml but low OH, were found to be older and have lower handgrip strength and lower lean tissue index (LTI) but not necessarily lower body mass index (BMI), compared to patients in groups B with BNP values below the cut-off, 500 pg/ml and high OH. Of the nine transplanted patients, eight were found in groups A and B.

Most patients were overhydrated before haemodialysis. When the relationship between OH and ultrafiltrated fluid volume during haemodialysis was investigated, participants in groups B and D were found to be at risk for chronic OH with excess fluid left in the body also after dialysis. In group B, the prescribed target weight was on average 1.1 kg above normal hydration, as defined by bioimpedance analysis. In group D, this discrepancy was + 1.4 kg.

### Bivariate correlations

As shown in Table 3 BNP correlated significantly and positively with age (*r* = 0.56, *p* < 0.001), CRP (*r* = 0.28, *p* = 0.027), OH (*r* = 0.29, *p* = 0.02) and all-cause mortality (*r* = 0.48, *p* < 0.001), and negatively with handgrip strength (*r* = -0.28, *p* = 0.032), LTI (*r* = -0.36, *p* = 0.003) and albumin (*r* = -0.49, *p* < 0.001). No correlation was found between BNP and CV-deaths (*p* = 0.36); the proportion of CV-deaths was evenly distributed between individuals with BNP above or below 500 pg/ml at baseline. All-cause mortality was associated with age, BNP, handgrip strength, LTI, CRP and also the Charlson comorbidity index (CCI), but not with OH.

A significant correlation between OH and sex was found, indicating that OH is more common in men. The lean tissue index (LTI) was significantly correlated with handgrip strength. Both LTI and handgrip strength were correlated with sex, with men having higher values than women and negatively correlated with all-cause mortality.

**Table 3:** Bivariate correlations between selected baseline clinical and biochemical characteristics and all-cause mortality

	Sex (men)	HGS (kg)	BMI (kg/m²)	LTI (kg/m²)	CRP (mg/L)	BNP (pg/ml)	OH (L)	Alb (mg/L)	All-cause mortality
Age (years)	-0,051	-0,273*	-0,077	-0,343**	0,028	0,562**	0,006	-0,260*	0,379**
Sex (men)		,540**	-0,089	0,365**	0,131	-0,051	0,324**	-0,071	-0,020
HGS (kg)			0,095	0,560**	-0,093	-0,277*	0,145	0,315*	-0,341**
BMI (kg/m²)				0,145	-0,136	-0,141	-0,124	0,034	-0,003
LTI (kg/m²)					-0,120	-0,363**	0,134	0,272*	-0,409**
CRP (mg/L)						0,276*	0,112	-0,452**	0,335**
BNP (pg/ml)							0,290*	-0,491**	0,476**
OH (L)								-0,232	-0,044
Alb (mg/L)									-0,233

BNP and CRP are log10 transformed, BMI; body mass index, BNP: brain natriuretic peptide; CRP: C-reactive protein; HGS: handgrip strength; LTI: lean tissue index; OH: overhydration. *p ≤ 0. 05, **p ≤ 0. 01

### Survival analyses (Kaplan‒Meier and Cox regression)

**Fig. 2:**
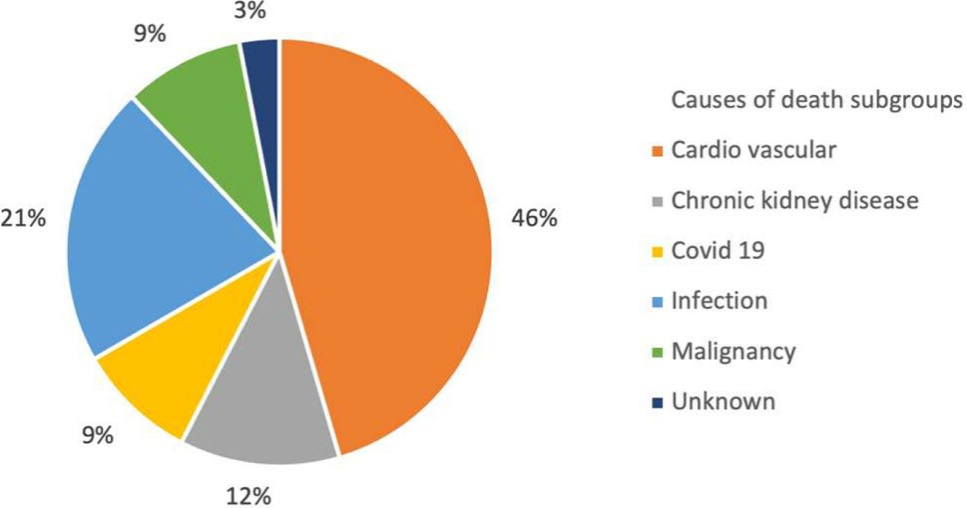
Sub-groups of causes of deaths expressed as percentages

**Fig. 3:**
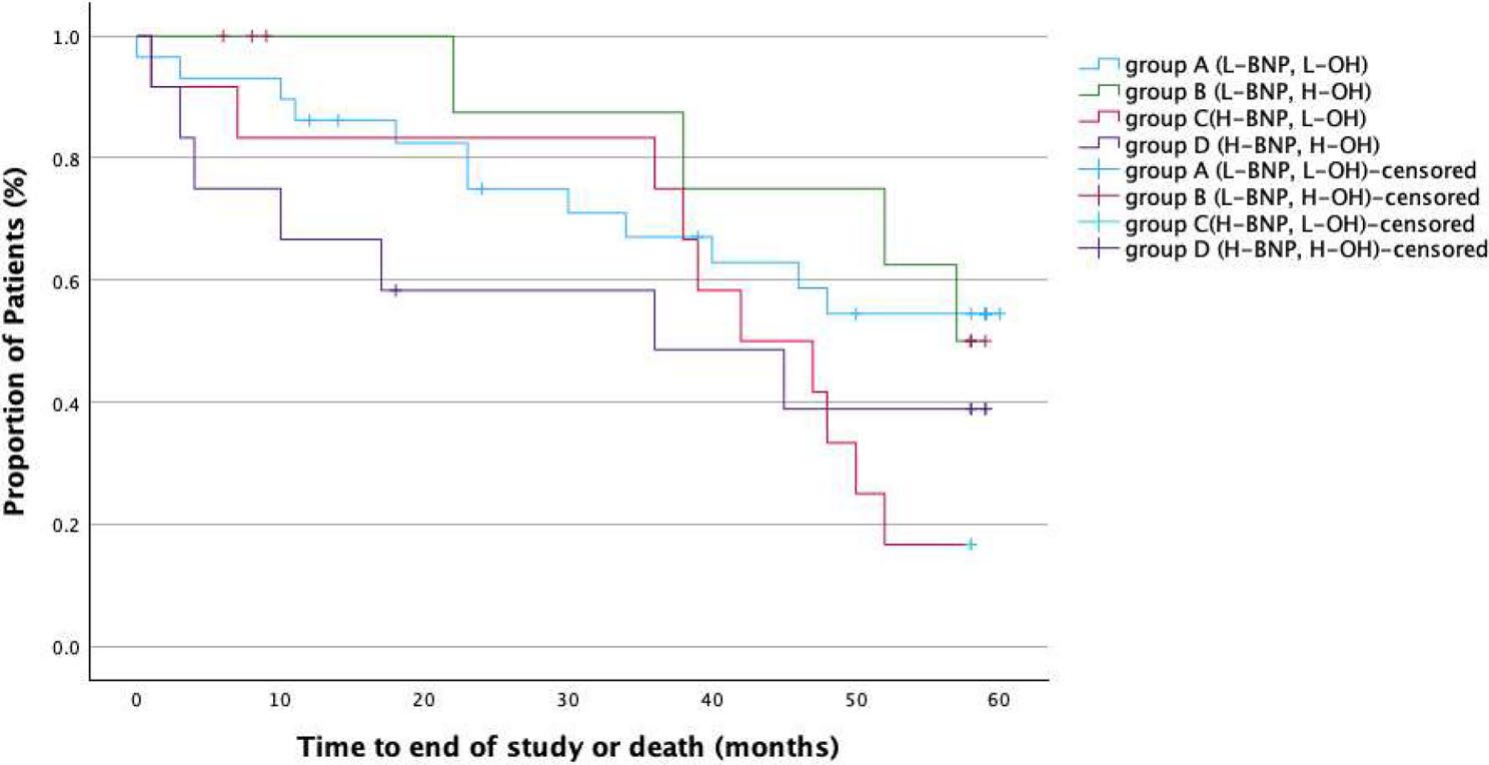
Kaplan‒Meier analysis of all-cause mortality in the four predefined groups A‒D. The figure depicts cumulative survival on y-axel and study observation time, which was up to 60 months, on x-axel. Transplanted patients were censored from survival analysis

**Fig. 4:**
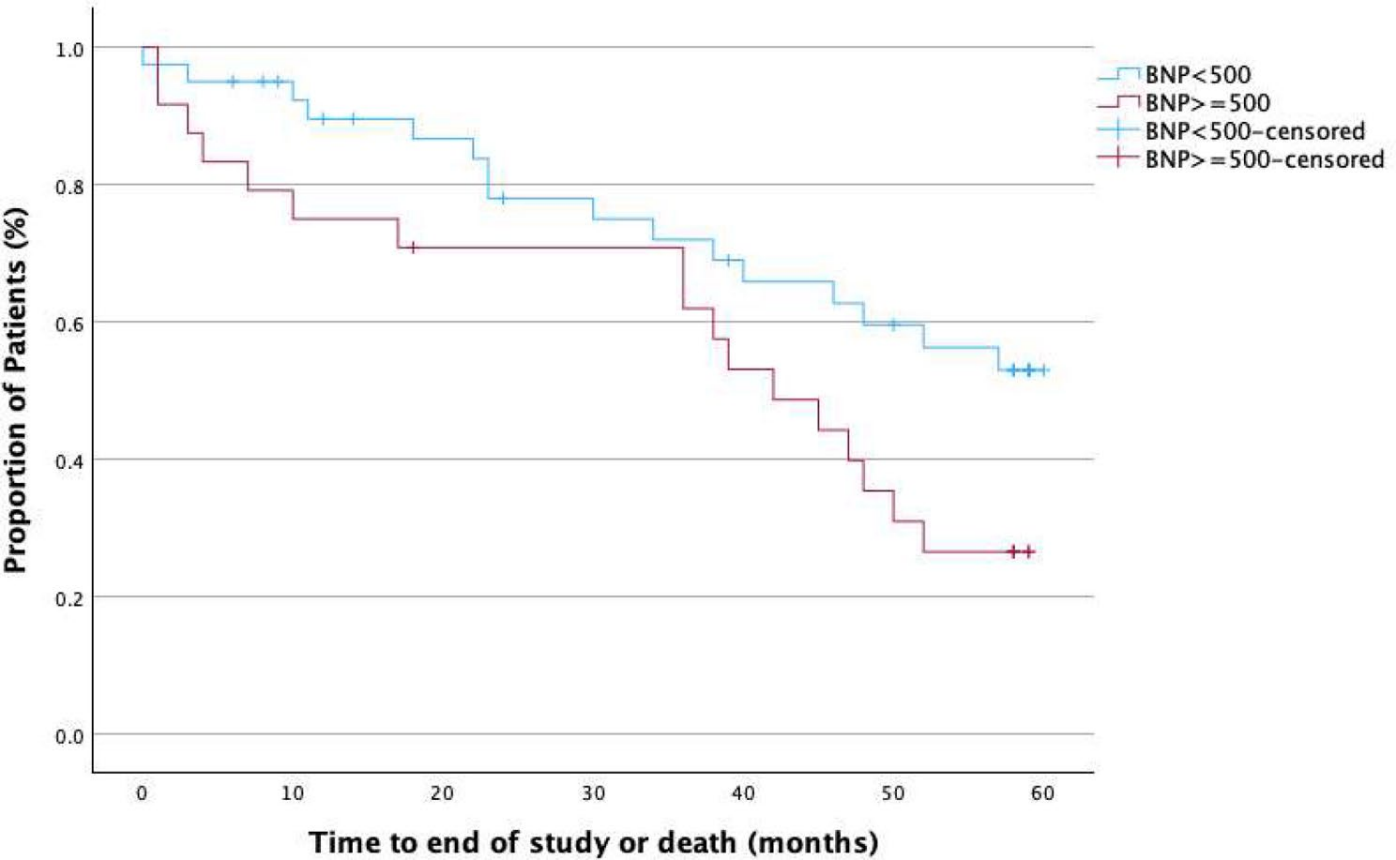
Kaplan‒Meier analysis of all-cause mortality in patients with a BNP value ≥ 500 or < 500 (pg/ml) (log-rank *p* = 0.016). The figure curve depicts the cumulative survival on the y-axel and the study observation time, which was up to 60 months, on the x-axel

Kaplan‒Meier analysis was used to analyse the time to event (all-cause mortality) in groups A–D, as previously defined. In total, 33 patients (52%) had died by the end of the study; 12 patients (41%) in group A, 4 (36%) in group B, 10 (83%) in group C and 7 (58%) in group D. Three patients had died due to a Covid-19 infection. 46% (*n* = 15) of the deaths were CV-deaths (Fig. 2). The results of the Kaplan‒Meier analysis for all-cause mortality in the four predefined groups A‒D are displayed in Fig. 3 (log-rank among all groups *p* = 0.062). As shown in Fig. 4, patients with BNP > 500 pg/ml, regardless of OH status (i.e., groups C and D), had a significantly higher mortality rate than patients with BNP ≤ 500 pg/ml (i.e., groups A and B), 71% vs. 40%, respectively, log-rank test *p* = 0.016.

In a univariable Cox regression model BNP was a significant predictor of all-cause mortality (HR 2.61, 95% CI: 1.34–5.11) (Table 4), but when BNP was adjusted for age, as shown in Model 2 in Table 4, the hazard ratio (HR) decreased from 2.61 to 1.99, and was no longer significant (HR 1.99, 95% CI: 0.94–4.21).

**Table 4:** Four models of univariable Cox regression analysis for all-cause mortality, adjusted for different risk factors

	Variable	HR	95% CI	*p* value
Model 1				
	BNP (pg/ml)	2.61	1.34–5.11	**0.005**
Model 2				
	BNP (pg/ml)	1.99	0.94–4.21	0.07
	Age (years)	1.03	1.00-1.07	0.07
Model 3a				
	BNP (pg/ml)	1.39	0.60–3.20	0.44
	Age (years)	1.03	0.99–1.07	0.11
	HGS (kg)	0.95	0.91–0.99	**0.023**
	CRP (mg/L)	2.72	1.41–5.24	**0.003**
Model 3b				
	BNP (pg/ml)	1.48	0.68–3.22	0.33
	Age (years)	1.04	1.01–1.08	**0.02**
	CCI	1.05	0.92–1.21	0.42
	CRP (mg/L)	2.04	1.13–3.90	**0.02**

BNP and CRP are log10 transformed. BNP: brain natriuretic peptide; CCI: Charlson comorbidity index; CRP: C-reactive protein

When BNP, in models 3a and 3b, was corrected for two additional co-variates correlating to mortality, the point estimate was 1.39 indicating that elevated BNP is an important predictor for all-cause mortality, but it was no longer statistically significant (*p* = 0.44). Instead, in this model, age, handgrip strength and CRP were all significant predictors of all-cause mortality (HR 1.03, 95% CI: 0.99–1.07; HR 0.95, 95% CI: 0.91–0.99; and HR 2.72, 95% CI: 1.41–5.24, respectively). Charlson comorbidity index (CCI), a well-known score for co-morbidities, correlated significantly to all-cause mortality in the bivariate correlation analysis, but when entered into the multivariable model replacing hand grip strength (HGS), the findings for BNP, age and CRP are similar, but in contrast to HGS, CCI did not contribute significantly to the model (HR = 1.05, 95% CI 0.92–1.21) as shown in Table 4. All VIFs were below 1.7, indicating low multicollinearity among predictors (Supplemental Table 1).

## Discussion

In this prospective observational study, the five-year all-cause mortality was high, but in line with national registry data from the Swedish renal registry [25]. High baseline BNP, CRP, and low handgrip strength were associated with all-cause mortality, at 5 years after assessment. There was a correlation between BNP and OH, but OH was not associated with all-cause mortality. Patients with elevated BNP levels demonstrated markedly greater mortality than patients with low BNP levels, highlighting the significant role of BNP as a prognostic biomarker. However, when adjusted for age, and factors associated with malnutrition and inflammation in a regression model, BNP was no longer a significant predictor of mortality.

The finding that elevated BNP levels may serve as a prognostic biomarker of all-cause mortality is in line with previous research. For example, a recent observational study reported that BNP values exceeding 500 pg/ml are strongly associated with increased mortality risk in a similar patient cohort [26]. Somewhat surprisingly, we found no association between BNP and CV death. In our study, as in many other studies, CRP was found to be a strong and independent predictor of all-cause mortality [27, 28]. Furthermore, a strong association between BNP and CRP was identified, and although no correlation was established between OH and CRP, the highest levels of CRP were observed in the subgroup of patients with the greatest degree of OH (group D). This observation is in line with the hypothesis that OH contributes to the inflammatory state observed in patients with kidney failure. In addition, haemodialysis per se introduces additional systemic inflammation beyond the baseline chronic inflammation seen in patients with kidney failure. Patients receiving haemodialysis are subjected to various factors contributing to inflammation, including the bio-incompatibility of dialysis membranes, catheter contamination, and the process of removing waste and excess fluids from the blood, which can activate the immune system and provoke inflammatory responses [29]. In populations with CKD with and without haemodialysis, BNP and CRP are regarded as risk markers for cardiovascular disease and CKD progression [30], and patients who present with inflammation and OH are at an increased risk of all-cause mortality compared with those without measurable signs of inflammation and OH [31]. This finding reinforces the importance of monitoring and managing both inflammation and fluid status in patients with kidney failure treated with haemodialysis, to mitigate and reduce cardiovascular risk and improve patient outcomes.

Previous studies have established a clear association between improved nutritional status, physical activity, increased quality of life, and reduced mortality risk, in patients with CKD [29]. In the present study, we identified handgrip strength as a robust predictor of all-cause mortality, demonstrating a stronger prognostic value than, e.g., the lean tissue index (LTI), as assessed by bioimpedance analysis. Handgrip strength, a simple yet reliable measure of voluntary muscle function, has been widely recognized as a cost-effective and potent indicator of physical function [32]. While primarily serving as a proxy for muscle mass and physical activity, handgrip strength is also strongly associated with nutritional status, comorbidities, quality of life, and all-cause mortality [33, 34].

Our findings are in keeping with prior observations in the general population, showing that handgrip strength is correlated with both age and sex [35]. Notably, the handgrip strength of our patients was slightly lower than that of the general population, with men exhibiting a mean handgrip strength of 30 kg and women 18 kg, compared with 31 kg and 20 kg, respectively, in an age-matched healthy population [35]. These results underscore the importance of frailty as a determinant of mortality in patients with CKD, emphasizing the need for early identification and intervention. The subgroup with the lowest handgrip strength (group C) had the highest mortality rate, reinforcing the association between reduced muscle strength and poor survival outcomes. Previous studies have demonstrated that higher body mass index (BMI), LTI, and handgrip strength are protective factors in patients undergoing haemodialysis [32, 36]. Interestingly, patients in group B, who exhibited high OH but low levels of BNP and who had the best nutritional status, indicated by higher handgrip strength, LTI, and albumin, had the lowest mortality rates in our study. This finding suggests that nutritional factors may play a more critical role in survival outcomes than OH alone does, suggesting that interventions aiming to improve nutritional status may be even more important than information about fluid restrictions. In this context it should also be noted that Charlson comorbidity index (CCI), a well-known score for co-morbidities and frailty, did not contribute significantly to the multivariable model when replacing hand grip strength (HGS). One interpretation of this finding could be that in patients with several co-morbidities and advanced disease, reduced muscle strength is more important than previous medical history for survival and HGS should therefore be further evaluated as a risk marker/factor.

In the general population, obesity is a well-recognized risk factor for cardiovascular disease and CKD, contributing to increased morbidity and mortality. However, this relationship does not hold universally across all populations. In patients with kidney failure and haemodialysis, observational studies have reported a phenomenon known as the “obesity paradox,” where a higher body mass index (BMI) is paradoxically associated with better survival outcomes [36, 37]. However, BMI may not be a precise marker of nutritional status or body composition in this population, as BMI does not distinguish between fat and muscle mass, which is crucial for understanding the health status of dialysis patients [36]. Indeed, weight loss accompanied by muscle gain is associated with improved survival, whereas weight gain combined with muscle loss is detrimental [37]. In our study, the group with the highest mortality (group C) did not have the lowest BMI, but the lowest lean tissue index (LTI), indicating that muscle mass depletion, rather than overall weight, may be a relevant predictor of mortality. This finding highlights the limitations of relying solely on BMI as a measure of health in patients with kidney failure and haemodialysis [38] and underscores the need for more advanced techniques to assess body composition, such as bioimpedance analysis or dual-energy X-ray absorptiometry (DXA), to provide a more nuanced understanding of how muscle and fat mass influence survival.

This study has several strengths, including its prospective design and long follow-up period, which increase the reliability of the findings but, there are also notable limitations. The small sample size limits the generalizability of the results to a broader population and reduces the statistical power to detect differences between subgroups. With 33 recorded events across four variables in the Cox regression models, the events-per-variable (EPV) ratio is 8.25, which is below the commonly recommended threshold of ≥ 10 to ensure robust regression estimates [39]. However, an EPV of ≥ 5 can still yield acceptable results [40]. Although the low EPV ratio may increase the risk of model instability in small samples, this study still provides valuable insights into understanding the role of BNP. Also, no evidence of multicollinearity was found. The results of the study adds to the body of evidence confirming the association between elevated BNP and mortality [16, 18], and even more important, sheds light on the close link between BNP and inflammation, nutritional status, but also handgrip strength and mortality [26, 32, 33]. The absence of repeated measurements for key variables, such as dialysis modality, inflammatory markers, fluid management and nutritional markers, means that temporal changes could not be assessed. At follow-up, we chose to add retrospective data on CCI to compensate for this, and in survival analyses, patients who received a kidney transplant were censored at the date for surgery. During the study period, there were no major changes in dialysis treatment recommendations at the clinic e.g., regarding online treatment with hemodiafiltration. Data were collected during the COVID-19 pandemic, which may have influenced mortality outcomes; however, COVID-19 was the cause of death in three of the 33 deaths only, suggesting a small direct impact on the study’s mortality findings.

## Conclusion

In conclusion, in this single-centre cohort of 64 patients with CKD and haemodialysis, BNP was correlated with all-cause mortality in univariable but not multivariable regression analyses, and OH was not found to correlate with all-cause mortality. Other clinical and biochemical factors, such as age, inflammation, and handgrip strength, were found to be more important factors associated with all-cause mortality than BNP. It is important to continue investigating the role of these factors and potential interventions in improving the prognosis and care of patients with kidney failure and receiving haemodialysis treatment. We hence recommend that these findings to be confirmed in larger studies.

## Electronic supplementary material

Below is the link to the electronic supplementary material.

Supplementary Material 1

## Data Availability

The data that support the findings of this study are not openly available due to reasons of sensitivity and are available from the corresponding author upon reasonable request.

## Abbreviations

BMI: Body mass index
BNP: Brain natriuretic peptide
CKD: Chronic kidney disease
CV: Cardiovascular
DXA: Dual-energy X-ray absorptiometry
EPV: Events-per-variable
FO: Fluid overload
LTI: Lean tissue index
OH: Overhydration
