# Supplementary material for: Brain natriuretic peptide and all-cause mortality in patients treated with haemodialysis
Source: BMC Nephrol. 2025 Jun 23;26:291. doi: 10.1186/s12882-025-04251-8 (PMC12186325; doi:10.1186/s12882-025-04251-8)
Supplement: Supplementary file 1 — Supplementary Material 1 [file 12882_2025_4251_MOESM1_ESM.docx]

# Supplementary material


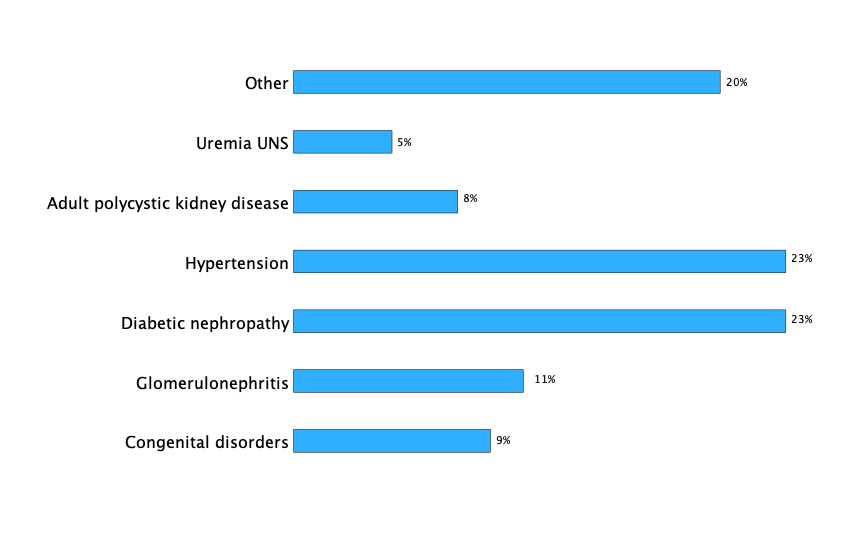


Supplemental figure 1. Kidney disease frequencies in all study participants, in percent.

Supplemental Table 1. Assement of multicollinearity

| **Variable** | **VIF** |
| --- | --- |
| BNP | 1.6 |
| Age | 1.5 |
| HGS | 1.1 |
| CRP | 1.2 |
| CCI | 1.2 |
| BNP: brain natriuretic peptide; CCI: Charlson comorbidity index; CRP: C-reactive protein VIF: Variance Inflation Factors. BNP and CRP are log10 transformed. | |
